# Hemoglobin A1c Serum Level Predicts 5-year Mortality in Patients with Cognitive Impairment

**DOI:** 10.1007/s40200-023-01303-4

**Published:** 2023-09-25

**Authors:** J. Dreier, E. Schernhammer, H. Haslacher, E. Stögmann, J. Lehrner

**Affiliations:** 1 Department of Neurology, Medical University of Vienna, Vienna, Austria; 2 Department of Epidemiology, Center for Public Health, Medical University of Vienna, Vienna, Austria; 3 Department of Laboratory Medicine, Medical University of Vienna, Vienna, Austria; 4 Neurologische Universitätsklinik, Allgemeines Krankenhaus, Währinger Gürtel 18-20, 1097 Vienna, Austria

**Keywords:** 5-year mortality, Alzheimer’s disease, MCI, SCD, Hemoglobin A1c, Neuropsychological battery

## Abstract

**Background:**

Subjective cognitive decline (SCD) and mild cognitive impairment (MCI) may occur as preclinical stages of Alzheimer's disease (AD), ultimately leading to dementia. Glycated hemoglobin A1c (HbA1c) is a diagnostic marker for diabetes mellitus and indicates mortality risk.

**Objectives:**

This university-based, exploratory retrospective study examined the impact of HbA1c serum level on 5-year mortality among individuals with cognitive impairment.

**Methods:**

Included were 1076 subjects aged at least 50 years who visited the Memory Outpatient Clinic of the Medical University of Vienna due to memory problems. Participants were diagnosed with SCD, MCI, or AD subsequent to neurological examination, standard laboratory blood tests, and neuropsychological testing. Survival was compared between diagnostic subgroups and with respect to HbA1c categories using log-rank tests based on Kaplan–Meier functions. The Neuropsychological Test Battery Vienna (NTBV) was dimensionally reduced, and a principal component analysis (PCA) was performed to further analyze results. Corresponding factor scores, HbA1c values, and baseline characteristics were included in Cox proportional hazards models to assess 5-year mortality risk.

**Results:**

During the observation period, 323 patients (30%) died at a mean age comparable between diagnostic subgroups (SCD 84.2 ± 10.1, MCI 81.2 ± 8.3, AD 82.2 ± 7.4 years). Individuals with normal serum HbA1c levels had significant advantages in survival within the MCI (12.9 ± .3 vs. 10.0 ± .8 years) and the AD subgroups (8.2 ± .4 vs. 5.5 ± .6 years), and metric HbA1c predicted 5-year mortality (HR 1.24).

**Conclusion:**

This study demonstrates an association between abnormal HbA1c serum levels and increased mortality.

## Background

In later life, cognitive performance declines as a part of normative aging processes [1]. In some individuals, however, a more pronounced deterioration can be detected, objectified and quantified with the aid of neuropsychological tests [2]. Due to an increasing awareness about dementia in the general population, the concept of subjective cognitive decline (SCD) has been established [3]. SCD refers to individuals' concerns about cognitive decline that cannot be objectified by neuropsychological testing [3, 4]. If cognitive deficits are manifested, but everyday life is fundamentally still manageable, the term mild cognitive impairment (MCI) is applied [5]. A more extensive cognitive decline and the resulting deficits and inabilities in everyday functions are clinical features of dementia [2], most commonly caused by Alzheimer's disease (AD) [6]. After a years-long asymptomatic preclinical phase, AD progresses toward an amnestic dementia syndrome [6]. MCI and SCD are considered preliminary stages that may occur in some, but not all, individuals suffering from AD. [3, 4, 6] Increased mortality rates among AD patients have been reported [6].

Glycated hemoglobin A1c (HbA1c) is created by non-enzymatic chemical reaction processes with glucose, whereby the resulting quantity correlates with the average blood glucose level [7]. Therefore, HbA1c serum level may be used as a diagnostic tool to detect diabetes mellitus (DM) [8]. It is considered a risk factor for all-cause mortality[9–11] and has been described as a predictor of cognitive decline [12–15]. Common underlying pathophysiological principles between AD and DM are being discussed [12, 13, 16]. Furthermore, HbA1c level was shown to influence both disease risk and progression [17, 18].

We hypothesize an increased 5-year mortality among cognitively impaired individuals with elevated HbA1c compared to normal serum levels. In the present study, we aim to investigate the relationship between HbA1c status and 5-year mortality in patients attending a memory outpatient clinic.

## Materials and Methods

This retrospective single-center analysis was based on data collected by the Department of Neurology of the Medical University of Vienna between November 1, 2004 and March 8, 2020. The HbA1 laboratory value was measured routinely from the beginning of the observation period. The study protocol of this analysis was approved by the ethics committee of the Medical University of Vienna under EK No. 2345/2019 and followed the principles of the Declaration of Helsinki.

### Participants

Study subjects visited the memory outpatient department due to self-reported memory problems or referrals by physicians. After undergoing neurological examination and neuropsychological testing, participants were assigned to an AD, MCI, or SCD subgroup. In accordance with Jessen et al., SCD classification criteria included (a) the presence of subjective cognitive deterioration as manifested by seeking of medical help for cognitive problems and (b) the current absence of any objectively measurable cognitive deficits (mean z-scores in each cognitive domain greater than -1.5 SD) [4, 19]. MCI was classified according to Petersen criteria, requiring a z-score of 1.5 SD below age and education-corrected norms [5, 19]. AD demented patients were diagnosed using the NINCDS-ADRDA criteria [2]. Furthermore, participants’ blood samples were obtained to measure HbA1c serum level. Those included in this study were individuals aged 50 years and above who completed at least the Mini Mental Status Examination (MMSE)[20] and had their HbA1c serum level measured from blood samples collected within ± one month of the examination date. Any conditions affecting cognition, such as stroke, head trauma, organ failure, psychiatric conditions that may cause pseudo-dementia or non-AD dementia, were exclusion criteria.

Participants’ sociodemographic data, neuropsychological testing results and diagnostic subgroup assignments were imported into an SPSS® file and listed by means of identification numbers. Participants were thus identified in the Allgemeines Krankenhaus Informations Management (AKIM), where the clinic records all services, to acquire their HbA1c blood test results. All available participant death data up to March 8, 2020, was gathered from the Research, Documentation and Analysis (RDA) system of the Medical University of Vienna. This system is updated annually and synchronized with the death data from Statistik Austria. In cases where there were no death data available, either from the RDA or the AKIM, it was concluded that the relevant participants were still alive at the end of study.

**Fig. 1:**
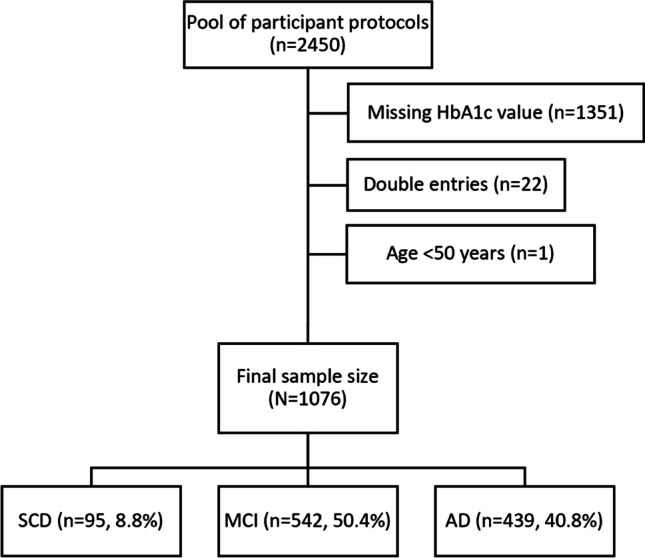
Flowchart with frequencies and corresponding percentages of diagnostic subgroups within the study sample (N = 1076). Abbreviations: HbA1c, glycated hemoglobin A1c; SCD, subjective cognitive decline; MCI, mild cognitive impairment; AD, Alzheimer's disease

After all data were imported, 2450 protocols were registered. During data cleaning, 1351 cases were removed due to missing HbA1c data. Furthermore, 22 duplicate entries and one case aged under 50 years had to be excluded. Figure 1 displays the the data screening process to obtain 1076 valid patient protocols that met all inclusion and exclusion criteria.

### HbA1c serum level

Blood samples for standard laboratory testing were provided by the Department of Neurology at the Medical University of Vienna. All samples were collected ± one month from neuropsychological assessment. HbA1c serum level was measured by the Clinical Institute for Laboratory Medicine of the General Hospital Vienna (AKH-Wien) using high-performance liquid chromatography (HPLC). The reference value in healthy subjects is 4.0–6.0 relative % and does not differ in gender; thus, reference values for men and women are not separated [21]. Values ranging from 5.7 to 6.49% indicate prediabetes [22]. Those 6.5% and above allow the diagnosis of diabetes mellitus to be established [23].

### Neuropsychological instruments / inventories

Neuropsychological testing was performed in the memory outpatient department of the Medical University of Vienna on each subject in one session. The Mini Mental State Examination (MMSE) was used as a screening tool for cognitive impairment. Healthy individuals may achieve up to 30 points; reduced scores correlate with the presence of cognitive impairment [20]. Symptoms of depression were assessed using the Geriatric Depression Scale short form (GDS-15) [24, 25]. Omitting physical factors that depend on age [26], this self- rating screening tool is suitable for the elder population as well as for cognitively impaired individuals [25, 26]. While up to four points are considered normal, scores of five points and above indicate mild, moderate, or severe depression. To assess their cognitive performance across multiple domains, participants underwent the Neuropsychological Test Battery Vienna short version (NTBV-15). This standardized clinical-use test kit was developed and validated to identify patients at risk for cognitive deterioration and to detect cases of dementia early on in a clinical setting. This inventory covers psycho-motor-speed, attention, language, memory, and executive function [27, 28]. The NTBV can be obtained from www.psimistri.com. Because of redundancy, MMSE results are not being further discussed or analyzed in this study.

### Statistical analysis

Descriptive and inferential statistical analyses were performed using IBM SPSS® 27 for Windows 10®. Metric variables are presented here as mean (*M)* ± standard deviation (*SD)*, minimum (min) and maximum (max). In cases of skewed distribution according to the nonparametric Kolmogorov–Smirnov and Shapiro–Wilk tests [29], the alternative measure median (*Mdn*) and interquartile range (IQR, 25–75%) are given.

In all analyses, the per-protocol approach was used, i.e., the available patient data were examined, and no imputations were made in case of missing values. Two-tailed statistical tests were performed, assuming an α = 5% significance level. Thus, results with *p* ≤ 0.05 are described as “significant”. To prevent type 1 error accumulation in the case of multiple testing, the significance level was adjusted according to Bonferroni using the equation α* = ($\frac{\alpha }{k}$). Kruskal–Wallis tests were used to compare dependent variables between the independent diagnostic subgroups, followed by Mann–Whitney U-tests for posthoc pairwise comparison in case of significance [29]. Survival rates and mean and median estimated survival times were calculated based on Kaplan–Meier (KM) functions, taking censored cases into account, with respect to the diagnostic subgroups and depending on the HbA1c category [30]. To address our primary research question, posthoc pairwise log-rank tests were conducted to assess differences between subgroups within the sample [31, 32]. Multivariate Cox proportional hazards models were used to calculate hazard ratios (HR) corresponding to the predictive value of metric and categorical HbA1c serum levels for 5-year survival. Covariates, including GDS-15 results, NTBV-15 factor scores, and demographic variables, were selected based on previous analysis. Predictors and covariates were added block-wise and simultaneously using the enter method [33, 34].

## Results

### HbA1c serum level

HbA1c % at admission was assessed close to neuropsychological testing; the mean time difference regarding cognitive testing was 0.1 ± 0.3 (min -0.9–max 1.0) months. HbA1c values showed a skewed distribution according to significant Kolmogorov–Smirnov testing in all diagnostic subgroups (*p*s < 0.05). Kruskal–Wallis testing revealed no significant difference in HbA1c serum level according to diagnostic subgroups, *H* (χ^2^, *df* 2) = 2.910, *p* = 0.233. However, when the ADA categories 4.0–5.69 (norm), 5.7– 6.49 (prediabetic), and ≥ 6.5% (diabetic) were applied in reference to the HbA1c serum level, Chi-square testing revealed a significant distribution difference, χ^2^ (*df* 4) = 10.853, *p* = 0.028, indicating heterogeneity of proportions. See Table 1 for descriptive statistics regarding metric and and categorical HbA1c level.

**Table 1:** Demographic baseline characteristics, HbA1c serum level, GDS-15 and z-scores according to the NTBV-15, including M ± SD, Mdn (IQR)

Parameter	Diagnostic subgroup			Total
	SCD	MCI	AD	
Age [years]	(n = 95) 67.5 (61.3; 74.7)	(n = 542) 70.6 (62.6; 76.9)	(n = 439) 76.8 (70.5; 81.0)	(n = 1076) 72.9 (65.1; 78.6)
Gender				
Male [n]	46 (9.6%)	249 (52.2%)	182 (38.2%)	477 (100%)
Female [n]	49 (8.2%)	293 (48.9%)	257 (42.9%)	599 (100%)
School attendance [years]	(n = 95) 12.0 (9; 15)	(n = 542) 11.5 (8; 16)	(n = 439) 9.0 (8; 12)	(n = 1076) 11.0 (8; 14)
Deceased [n]	9 (9.5%)	118 (21.8%)	196 (44.6%)	323 (30.0%)
Age at death [years]	(n = 95) 87.5 (74.0; 93.1)	(n = 542) 82.6 (75.7; 87.9)	(n = 439) 82.7 (77.2; 88.0)	(n = 1076) 82.7 (77.1; 88.0)
HbA1c [fraction %]	(n = 95) 5.5 (5.3; 5.8)	(n = 542) 5.6 (5.4; 5.9)	(n = 439) 5.6 (5.4; 5.9)	(n = 1076) 5.6 (5.4; 5.9)
Norm [n]	56 (58,9%)	281 (51,8%)	222 (50,6%)	559 (52,0%)
Prediabetic [n]	35 (36,8%)	211 (38,9%)	156 (35,5%)	402 (37,4%)
Diabetic [n]	4 (4,2%)	50 (9,2%)	61 (13,9%)	115 (10,7%)
GDS-15 [value]	(n = 93) 2.0 (1; 4)	(n = 523) 3.0 (1; 6)	(n = 328) 3.0 (1; 5)	(n = 944) 3.0 (1; 6)
NTBV-15 factor z-scores				
F1 *Vigilance*	(n = 95) .557 ± .504	(n = 523) .231 ± .740	(n = 284) -.611 ± 1.230	
F2 *Memory*	(n = 95) .739 ± .588	(n = 523) .284 ± .856	(n = 284) -.770 ± .889	
F3 *Executive functions*	(n = 95) .340 ± .684	(n = 523) .089 ± .980	(n = 284) -.278 ± 1.060	
F4 *Verbal comprehension*	(n = 95) .568 ± .697	(n = 523) .096 ± .942	(n = 284) -.368 ± 1.060	

Abbreviations: SCD, subjective cognitive decline; MCI, mild cognitive impairment; AD, Alzheimer's disease; HbA1c, glycated hemoglobin A1c; GDS-15, Geriatric Depression Scale short version, containing 15 items; NTBV-15, Neuropsychological Test Battery Vienna short version, containing 15 items

### NTBV-15 principal component analysis

The NTBV-15 analysis comprised the data protocols of 902 individuals who completed neuropsychological testing. According to the computed KMO value of 0.90, the variance in response behavior on the NTBV-15 subscales could be explained using fewer dimensions (factors) [35]. An explorative principal component factor analysis (PCA) was employed to achieve dimensional reduction. The subsequently performed Kaiser’s Varimax orthogonal rotation converged at the 11th iteration [35]. The communality h_i_^2^ (≤ 1), the row sum of the squared loadings of an item across the extracted factors, the eigenvalue λ (≥ 1), and the column sum of the squared loadings per factor across the items are relevant indicators to interpret the results of a factor analysis [33, 36]. Based on the function of the eigenvalue λ in the scree plot, four factors describing the cognitive structure of the subjects were determined. Thus, a cumulative 77.0% of variance could be explained, while the communality h_i_^2^ reached fairly high values for all subtests [33]. The resulting factor scores were mutually uncorrelated (*r* = 0) and z-standardized (μ = 0, σ = 1). As they were weighted and unaffected by the polarity sign of the loadings, they were suitable for further analysis without loss of information [37]. The item loadings of the NTBV-15 subtests within the four computed factors, including the communality and eigenvalue, are displayed in Table 2.

**Table 2:** Factor loadings of the NTBV-15 subtests and explained variance when computing four factors

NTBV-15 subtest	Dimension				Communality h_i_^2^
	F1	F2	F3	F4	
AKT time	-.793	-.235	-.257	-.079	.76
Symbols counting (c.I.)	-.781	-.172	-.191	-.144	.70
Interference time (c.I.)	-.779	-.224	-.170	-.342	.80
Interference total score / time (c.I.)	.738	.267	.142	.343	.76
AKT total score / time	.738	.305	.303	.147	.75
Psychomotor processing speed (TMT A)	-.678	-.242	-.398	-.190	.71
VSRT delayed recall	.219	.865	.152	.178	.85
VSRT total recall	.322	.857	.149	.208	.90
VSRT immediate recall	.291	.801	.091	.154	.76
VSRT recognition	.114	.780	.163	.088	.66
Maze total score / time	.407	.211	.766	.106	.81
Maze time	-.505	-.194	-.757	-.123	.88
PWT letter f	.414	.170	-.034	.729	.73
Naming (BNT)	.062	.232	.516	.665	.77
SWT Animals	.429	.429	.209	.559	.72
Eigenvalue (λ)	4.42	3.44	1.95	1.74	Σ 11.55
Explained variance	29.5%	22.9%	13.0%	11.6%	77.0%

Abbreviations: NTBV, Neuropsychological Test Battery Vienna; AKT, Alters Konzentrations Test; TMT A,; BNT, Boston Naming Test; PWT, Phonematic Verbal Fluency Test; VSRT, Verbal Selective Reminding Test

A pattern of cognitive structure can be assumed from performances on subtests that load mainly on one factor. Considering the group of subtests loading on the same factor, it is proposed to name the dimensions F1 *Attention*, F2 *Memory*, F3 *Executive functions* and F4 *Verbal fluency and naming*, in alignment with a previous publication on the NTBV [38]. Conducting a Levene test revealed heterogeneity of variance among the four factor scores in the three diagnostic subgroups (*p* < 0.001). Welch ANOVAs were performed to compare the performance between the three diagnostic subgroups, taking the Bonferroni correction (α* = 0.0125) into account [34]. This revealed significant differences for all four factors (F(2, 899) = 99.654; F(2, 899) = 184.562; F(2, 899) = 19.296; F(2, 899) = 40.167; *p*s < 0.001), with effect sizes ranging from small (0.04; Executive functions) to large (0.29; Memory). Posthoc pairwise analyses using Games-Howell procedure[29] and considering the Bonferroni correction (α* = 0.0167), revealed a hierarchy among all four factors SCD > MCI > AD (*p*s < 0.01).

### Assessing 5-year survival

**Fig. 2:**
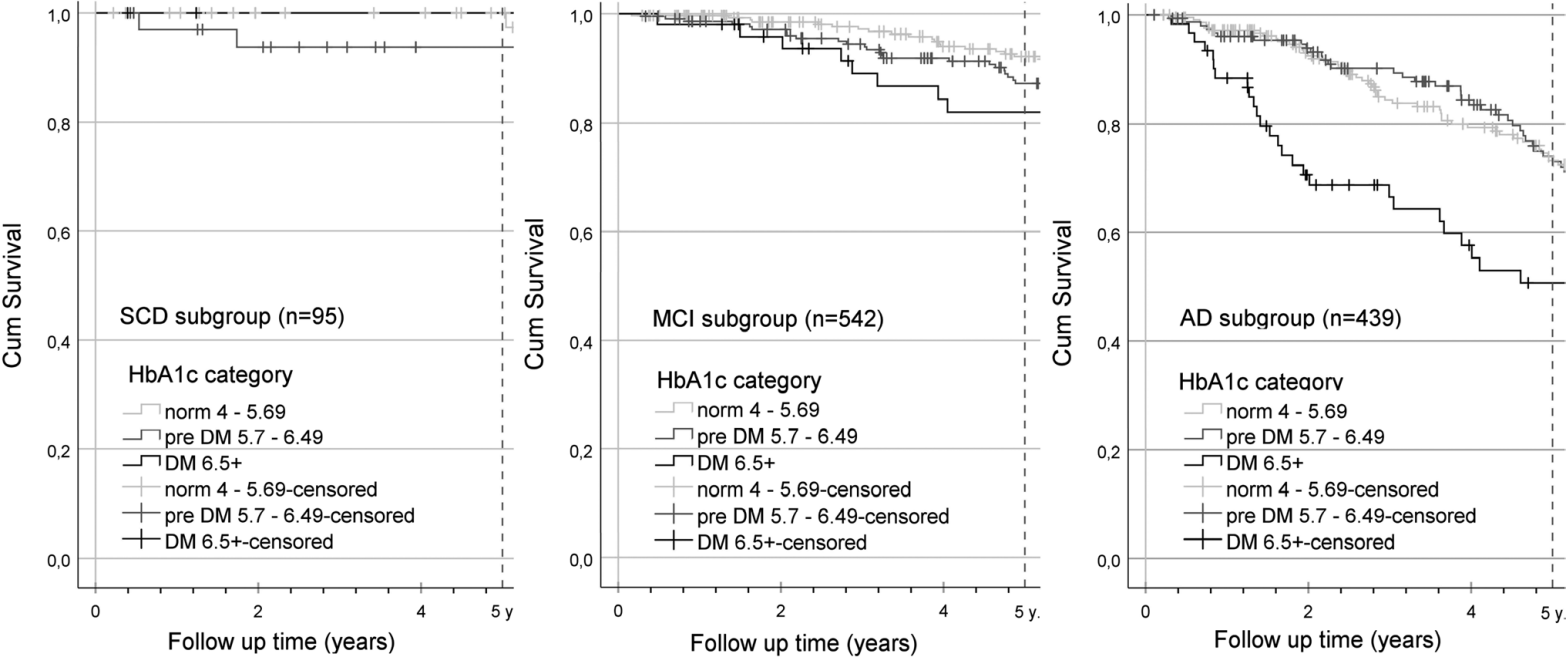
KM survival functions regarding HbA1c % categories, considering the diagnostic subgroups (SCD n = 95, MCI = 542, AD = 439), taking 5- survival likelihood into account. Abbreviations: SCD, subjective cognitive decline; MCI, mild cognitive impairment; AD, Alzheimer's disease; HbA1c, glycated hemoglobin A1c; DM, diabetes mellitus

According to Kaplan–Meier (KM) survival functions, and considering the information of deceased and censored participants within the follow-up time, the 5-year survival rates of the diagnostic subgroups could be assumed to be 97.6% for SCD, 89.2% for MCI and 70.0% for AD. Overall, significant differences in survival times were observed for the three diagnostic subgroups using log-rank testing, χ^2^(2) = 142.608, *p* < 0.001 (n = 1076). Posthoc pairwise comparisons for median and mean survival regarding the three diagnostic subgroups using log-rank tests revealed for SCD (*Mdn* = 15.2, *M* = 13.9) vs. MCI (*M* = 12.2) years, χ^2^(1) = 6.764, *p* = 0.009, SCD vs. AD (*Mdn* = 7.2, *M* = 8.0) years, χ^2^(1) = 48.110, *p* < 0.001 and MCI vs. AD, χ^2^(1) = 112.681, *p* < 0.001 either significant differences for overall survival after study entrance. Subsequently, KM functions regarding HbA1c categories were calculated for all three diagnostic subgroups separately, as shown in Fig. 2.

Log-rank tests and posthoc pairwise comparisons of the survival functions, taking the Bonferroni adjustment (α* = 0.0167) into account, revealed an advantage in survival time for the normal HbA1c category compared to the diabetic category within the MCI subgroup (*M* = 12.92 vs. 10.01 years, χ^2^(1) = 12.224, *p* < 0.001). In the AD subgroup, both normal and prediabetic HbA1c serum levels showed an advantage in median survival time over the diabetic category (*Mdn* = 8.06 vs. 5.62 years, χ^2^(1) = 16.193, *p* < 0.001 and 7.84 vs. 5.62 years, χ^2^(1) = 14.963, *p* < 0.001). It should be noted that this function could not be estimated principally for the SCD subgroup, and mean estimates are given here instead of median survival for the MCI subgroup, as the latter could only be calculated if the KM-function declined below 50%.

Table 3 displays the mean and median estimates for the MCI and AD groups.

**Table 3:** Mean and median estimates for survival time (years) from study entrances regarding the MCI and AD diagnostic subgroups, considering HbA1c categories

	Mean				Median			
MCI	95% CI				95% CI			
	Estimate	*SE*	LB	UB	Estimate	*SE*	LB	UB
HbA1c norm	12.92	.30	12.33	13.52	-	-	-	-
Pre-DM	11.82	.38	11.09	12.56	-	-	-	-
DM	10.01	.77	8.50	11.53	12.42	3.94	4.71	20.14
Overall	12.21	.23	11.75	12.67	-	-	-	-
Mean					Median			
AD	95% CI				95% CI			
	Estimate	*SE*	LB	UB	Estimate	*SE*	LB	UB
HbA1c norm	8.21	.38	7.46	8.96	8.06	.50	7.08	9.03
Pre-DM	8.40	.45	7.52	9.28	7.84	.52	6.83	8.85
DM	5.50	.60	4.32	6.68	5.62	1.11	3.44	7.79
Overall	8.01	.28	7.46	8.56	7.20	.40	6.42	7.99

Abbreviations: MCI, mild cognitive impairment; HbA1c, glycated hemoglobin A1c; DM, diabetes mellitus; AD, Alzheimer’s disease

To estimate the 5-year survival probability within the sample, multivariate analyses were performed using Cox proportional hazards models, taking follow-up time into account. Considering predictors and covariates, there were 859 complete records available, including 96 events and 763 censored cases, in the observation period. Predictors were added in a blockwise, hierarchical approach, using the enter method. In a first step (model I), we assessed metric HbA1c % in the first block, followed by sociodemographic variables such as age, gender, and years of education in the second block, depressiveness (GDS-15) in the third block, and the four cognitive domains according to the NTBV-15 in the fourth block as predictors for the 5-year mortality criterion. The summarized results of the final model step are shown in Table 4.

**Table 4:** Cox proportional-hazards model I including all four blocks for the criterion “5-year mortality” (n = 859 cases with complete data protocols)

Predictor NTBV domain/ covariates	*B*	*SE*	Wald χ^2^	*df*	*p*-value	HR	95% HR	
							LB	UB
HbA1c (% metric)	.213	.091	5.471	1	.019^*^	1.238	1.035	1.480
age (years at testing)	.077	.015	25.843	1	< .001^**^	1.080	1.048	1.112
gender (0 male, 1 female)	-.770	.222	12.044	1	.001^**^	.463	.300	.715
education (school years)	-.037	.030	1.507	1	.220	.964	.908	1.022
GDS-15	.092	.031	9.106	1	.003^**^	1.097	1.033	1.165
F1 *Vigilance*	-.279	.101	7.630	1	.006^**^	.757	.621	.922
F2 *Memory*	-.377	.125	9.091	1	.003^**^	.686	.537	.876
F3 *Executive functions*	-.036	.103	.126	1	.723	.964	.788	1.179
F4 *Verbal comprehension*	-.195	.101	3.692	1	.055	.823	.675	1.004

Abbreviations: NTBV-15, Neuropsychological Test Battery Vienna short version, containing 15 items; HbA1c, glycated hemoglobin A1c; GDS-15, Geriatric Depression Scale short version, containing 15 items

^**^p ≤ .01, ^*^p ≤ .05

Model I results indicated significant explanatory value for metric HbA1c % (HR 1.24; *p* = 0.019), for age at testing, for male gender (*p*s < 0.001), and for GDS-15 depressiveness (*p* < 0.01) as predictors of 5-year mortality, but not of years of schooling. Considering the NTBV-15 factors, the inverted values (1/HR) of the hazard ratios for *Attention* and *Memory* showed an increased mortality risk in proportion to poorer test performance (*p*s < 0.01), whereas *Executive functions* and *Verbal fluency and naming* had no significant explanatory value predicting mortality.

In a second step (model II), the same Cox model was applied, but in block I, categorical HbA1c serum levels (HbA1c categories {0} *norm*, {1} *prediabetic*, {2} *diabetic*) were evaluated as dummy-coded predictors, considering *norm* as the reference category. According to block I analysis, *diabetic* HbA1c showed significant evidence (HR 2.73; *p* < 0.001) of increased relative risk for 5-year mortality, while no significant explanatory value could be derived for prediabetes. Subsequently, the second, third, and fourth blocks were added in the same manner as for model I. The summarized results of model II analysis, including all covariates of all four blocks, are shown in Table 5.

**Table 5:** Cox proportional-hazards model II including all four blocks for the criterion “5-year mortality” (n = 859 cases with complete data protocols)

Predictor NTBV domain/ covariates	*B*	*SE*	Wald χ^2^	*df*	*p*-value	HR	95% HR	
							LB	UB
HbA1c			2.571	2	.277			
Prediabetic vs. norm	.098	.229	.181	1	.670	1.102	.704	1.728
Diabetic vs. norm	.467	.294	2.523	1	.112	1.594	.897	2.836
age (years at testing)	.078	.015	26.116	1	< .001^**^	1.081	1.049	1.114
gender (0 male, 1 female)	-.759	.223	11.621	1	.001^**^	.468	.302	.724
education (school years)	-.039	.030	1.681	1	.195	.962	.906	1.020
GDS-15	.095	.030	9.616	1	.002^**^	1.099	1.035	1.167
F1 *Attention*	-.297	.100	8.830	1	.003^**^	.743	.611	.904
F2 *Memory*	-.387	.125	9.581	1	.002^**^	.679	.531	.868
F3 *Executive functions*	-.037	.102	.132	1	.716	.964	.789	1.177
F4 *Verbal fluency and naming*	-.195	.103	3.564	1	.059	.823	.672	1.007

Abbreviations: NTBV-15, Neuropsychological Test Battery Vienna short version, containing 15 items; HbA1c, glycated hemoglobin A1c; GDS-15, Geriatric Depression Scale short version, containing 15 items

^**^p ≤ .01, ^*^p ≤ .05

According to full model II analysis, *diabetic* long-term blood glucose HbA1c no longer showed significant explanatory value, but indicated a trend as a categorical predictor (HR 1.59), whereas significant explanatory values for the relative risk of mortality within 5 years could be assumed (*p*s < 0.01) for increasing age (HR 1.08), male gender (inverted HR 2.14) and GDS-15 depressiveness (HR 1.10). Furthermore, no significant contribution could be derived for school attendance. Considering the NTBV-15 domains, an inverted hazard ratio HR was determined (*Attention* 1.35 and *Memory* 1.47), showing an increasing relative risk of mortality in proportion to poorer test performance (*p*s < 0.01), whereas no significant relative risk of mortality was found for *Executive functions* and *Verbal fluency and naming*.

## Discussion

By the end of the study, 323 (30%) deaths had been recorded. Survivors were treated as censored cases in the context of Kaplan–Meier plots. With a median age of 72.9 years at study entry, overall median survival was 13.4 years. This is similar to the proposed median further life expectancy of 12.1 years and 14.4 years, separated for men and women among the same-aged Viennese general population, according to 2021 mortality tables [39]. The AD subgroup (*Mdn* 76.8 years old) showed a 7.2-year median survival, which was considerably shorter than the expected 9.8 and 11.6 further live-years in peer males and females [39]. The literature reports relatively similar survival periods among AD patients: Ganguli etl al [40]. found a mean survival of 5.9 years from the age of 80.2, while Williams et al.[41] observed a mean survival time of 8.5 years after onset at 74.6 years. Other authors observed a survival from 4 to 8 years among 65-year olds from the point of diagnosis [6]. The survival function did not decline below 50% for MCI, so no median survival time could be calculated, and mean estimates were given instead. Within the SCD subgroup, the observed median survival time was similar to further live-years expectancy among Viennese peers [39].

In pairwise comparisons regarding survival time using log rank tests, the SCD subgroup held an advantage over MCI and AD participants. However, all three diagnostic subgroups shared a similar median age at death, while the age at study entry showed a hierarchy in reverse order. This observation reflects the proposed stadial course of AD with SCD and MCI as preclinical stages that may occur years prior to the demential syndrome in comparably younger individuals [3, 4]. When survival curves related to HbA1c categories in the three diagnostic subgroups were compared separately, a survival advantage for *norm* over *diabetic* became apparent in the MCI subgroup. Among AD patients, comparisons revealed an advantage in survival time for both the *norm* and the *prediabetic* category over *diabetic* HbA1c values. In a finding that supported our primary hypothesis, we observed increased mortality among cognitively impaired individuals with elevated compared to normal HbA1c serum levels. Meta-analyses examining the association between HbA1c and mortality in different settings resulted in similar findings [8–11].

Furthermore, the Cox proportional hazards model assessing 5-year mortality risk indicated predictive value for metric HbA1c serum level among patients at different stages of cognitive decline. This result could be expected, as the literature reports an increased risk of mortality for both Alzheimer’s disease (AD) dementia patients and non-demented individuals with elevated HbA1c serum level [8, 42]. However, when HbA1c serum level categories *norm*, *prediabetic* and *diabetic* were applied, none of them predicted 5-year mortality. According to the present data, mortality generally increases with HbA1c; however, the category *diabetic* is not indicative. Bi-directional effects between HbA1c and mortality in diabetic individuals have been described in the literature. Accordingly, mortality risk increases with both elevated and very low HbA1c serum levels [10]. More specifically, in healthy subjects, mortality increases at an HbA1c serum level > 6%, whereas in diabetic patients, mortality increases > 7.5% [8, 10].

Cox proportional hazards model results also indicated predictive value for age, male gender, and GDS-15 severity, as well as the domains F1 *Attention* and F2 *Memory* regarding mortality. These findings are consistent with those in a previous publication on the NTBV and have been discussed there to a greater extent [38].

The retrospective design of the present study limits its informative value. Only 859 out of 1076 participants (79.8%) completed the NTBV-15 and thus were suitable for our Cox proportional hazards models. In the absence of a healthy control group, relevant study parameters were compared with regard to the three diagnostic subgroups, and survival expectancy estimates in age peers were given to put the results into perspective. Furthermore, data on participants’ intake of antidiabetic medication were not available; distorting effects on HbA1c serum levels cannot be excluded. In addition, modified target values for HbA1c, which are derived from observations regarding mortality in diabetic individuals, could not be taken into account [18, 23].

In future studies, data on participants’ metabolic status and their antidiabetic drug intake should be collected during outpatients’ visits to examine relations between HbA1c serum level and mortality considering specific target values.

To conclude, the observed survival time among AD diagnosed individuals is consistent with previous studies and reduced compared to peers. Kaplan–Meier functions indicate an survival advantage for *normal* over *diabetic* HbA1c serum level in patients with MCI and AD in the present study. Cox proportional hazards model results show predictive value indicating 5-year mortality for metric but not categorized HbA1c % serum level among patients with cognitive decline. Female gender is associated with higher survival probability, while age, depressive symptoms, and poor results in neuropsychological testing increase risk of mortality. Our findings underscore the potential importance of monitoring HbA1c levels as a part of comprehensive care for individuals with cognitive deterioration.

## Data Availability

The datasets generated during and analyzed during the current study are available from the corresponding author on reasonable request.
